# Correction to “Effect of PDO Facelift Threads on Facial Skin Tissues: An Ultrasonographic Analysis”

**DOI:** 10.1111/jocd.16596

**Published:** 2024-10-18

Lots TCC and Moraes P. Effect of PDO facelift threads on facial skin tissues: An ultrasonographic analysis. *J Cosmet Dermatol*. 2023; 22: 2534–2541. doi:10.1111/jocd.15761

Dr. Paulo Moraes, Scientific Coordinator at the European Face & Body Institute, should have been included in the author list. The contributorship of Dr. Moraes has been confirmed by the European Face & Body Institute. Telma Cerbino Cintra Lots was not available for confirmation.

The corrected author list should read as follows: Telma Cerbino Cintra Lots, Universidad Europea Miguel de Cervantes, Valladolid, Spain, dra.telmacclots@gmail.com, Dr. Paulo Moraes, European Face and Body Institute, moraes@europeanfaceinstitute.com.

We apologize for this error.

